# Absence of Association Between Glaucoma and Stroke Risk: Insights From a Cross‐Sectional Study and a Two‐Sample Mendelian Randomization Study

**DOI:** 10.1155/joph/5492641

**Published:** 2026-05-21

**Authors:** Yanan He, Qingke Shi, Dandan Chang, Yanshan Hu, Yulong Ma, Likai Shi, Hang Guo

**Affiliations:** ^1^ Department of Anesthesiology, The First Medical Center of Chinese PLA General Hospital, Beijing, 100853, China, 301hospital.com.cn; ^2^ Department of Anesthesiology, The Ninth Medical Center of Chinese PLA General Hospital, Beijing, 100101, China; ^3^ Department of Anesthesiology, The Seventh Medical Center to Chinese PLA General Hospital, Beijing, 100700, China; ^4^ The Second School of Clinical Medicine, Southern Medical University, Guangzhou, 510282, China, fimmu.com

**Keywords:** causal association, genetic analysis, glaucoma, Mendelian randomization, stroke

## Abstract

**Objective:**

Observational studies have reported an association between glaucoma and stroke risk; however, whether this relationship reflects causality remains unclear. This study aimed to comprehensively evaluate the association between glaucoma and stroke by integrating population‐based cross‐sectional analysis with Mendelian randomization (MR) to assess both observational and genetic evidence.

**Methods:**

A cross‐sectional analysis was conducted using data from the National Health and Nutrition Examination Survey (NHANES) 2005–2008 cycles. Multivariable logistic regression models were applied to examine the association between glaucoma and stroke after adjustment for demographic, socioeconomic, and cardiovascular risk factors. In parallel, a two‐sample MR analysis was performed using summary statistics from large‐scale genome‐wide association studies, evaluating overall glaucoma as well as primary open‐angle and neovascular glaucoma in relation to stroke and ischemic stroke subtypes. Causal estimates were primarily derived using the inverse‐variance weighted (IVW) method, with multiple sensitivity analyses to assess robustness.

**Results:**

In the NHANES analysis (*n* = 1312), glaucoma was not significantly associated with stroke in crude or fully adjusted models. In MR analyses, genetically predicted glaucoma showed no evidence of a causal association with stroke or ischemic stroke subtypes, including large artery atherosclerotic, cardioembolic, and small vessel stroke. These null findings were consistent across MR‐Egger, weighted median, and weighted mode methods. Sensitivity analyses accounting for heterogeneity, pleiotropy, and outlier instruments did not materially alter the results.

**Conclusion:**

Using complementary observational and genetic approaches, this study found no evidence supporting a causal relationship between glaucoma and stroke, providing new insights into their genetic epidemiology.

## 1. Introduction

Stroke, an acute cerebrovascular disease, has emerged as a leading contributor to increased disability‐adjusted life years (DALYs) and mortality globally [1]. Epidemiologically, it is estimated that annually there are around 12.2 million new stroke cases, contributing to approximately 143 million DALYs and resulting in 6.55 million deaths [2]. Strokes are categorized into ischemic and hemorrhagic types, with ischemic strokes further classified into large artery atherosclerotic, cardioembolic, and small vessel occlusion strokes. Beyond causing significant physical and cognitive impairments, stroke imposes a substantial economic burden on patients and their families. Despite advancements in treatments such as thrombolysis, antiplatelet therapy, and mechanical thrombectomy, the recurrence risk and long‐term disabilities remain high for many patients [3]. The incidence of stroke is rising, notably affecting younger populations, and is linked to multiple risk factors, including hypertension and diabetes. However, other potential etiologies that could contribute to stroke development are less explored, which hinders advancements in prevention and treatment strategies. Recent work further highlights the heterogeneity of stroke biology and outcomes and the need for refined etiologic and mechanistic understanding [4, 5].

In recent years, the link between glaucoma and stroke risk has garnered attention, particularly considering glaucoma as an exposure factor. Glaucoma, a major cause of blindness worldwide, primarily includes types such as primary open‐angle and neovascular glaucoma. Early findings indicate that stroke incidence in glaucoma patients significantly exceeds that in the general population [6]. Accumulating clinical observational studies and meta‐analyses suggests an elevated stroke risk among patients with glaucoma, yet reported effect sizes often attenuate after adjustment for vascular comorbidities and other confounders, raising concerns about residual confounding and bias. Observational studies highlight this association; for instance, a landmark case–control study reported a significantly higher stroke incidence in patients with open‐angle glaucoma compared to those without glaucoma [7]. A recent observational study also found a significant correlation between glaucoma and increased stroke risk (odds ratio [OR] = 5.278, 95% confidence interval [CI]: 1.094–25.462) [8]. Moreover, a study observed that the cumulative incidence of ischemic stroke was 15.6% higher in patients with neovascular glaucoma than in the control group [9]. Although these studies underscore a notable correlation between various types of glaucoma and stroke, confounding factors such as patient age, lifestyle, and comorbid conditions often complicate the substantiation of a causal link.

Critically, conventional observational designs cannot fully exclude unmeasured confounding, selection bias, and reverse causation (including differential healthcare contact and comorbidity detection). To address this causal gap, we conducted an observational analysis using NHANES data [10] and applied two‐sample Mendelian randomization (MR), which uses genetic variants as instrumental variables to strengthen causal inference and reduce confounding [11, 12]. Recent MR studies in other disease contexts further illustrate the utility of this approach for evaluating putative causal links when randomized trials are infeasible [11, 12]. This study aims to determine whether glaucoma causally influences stroke risk. By triangulating real‐world epidemiologic evidence with genetic instruments and by evaluating glaucoma and stroke at the subtype level, we seek to clarify whether clinically observed associations reflect a causal relationship. Resolving this question has direct clinical implications for risk stratification and comorbidity management. Clarifying this relationship helps inform clinical practice. A confirmed causal link from our genetic evidence would position glaucoma as a relevant factor for stroke risk assessment, potentially justifying enhanced preventive measures. In contrast, evidence against causality would reinforce that clinical management should prioritize aggressive control of shared vascular risk factors such as hypertension and diabetes over attributing independent stroke risk to glaucoma.

## 2. Materials and Methods

### 2.1. Cross‐Sectional Study

#### 2.1.1. Study Design and Participants

This study employed a complementary analytical framework to evaluate the relationship between glaucoma and stroke. The two‐sample MR analysis serves as the primary method for causal inference, leveraging genetic instruments to minimize confounding. In parallel, a cross‐sectional analysis using NHANES data provides a real‐world observational context, reflecting associations as measured in a population‐based survey with its inherent limitations.

**FIGURE 1:**
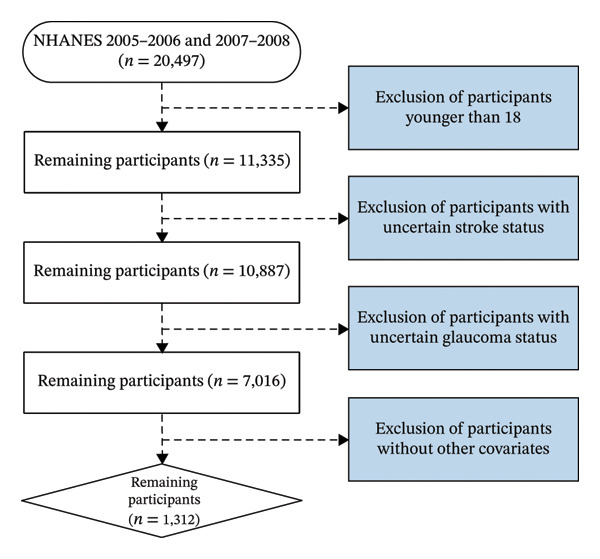
Flowchart for inclusion and exclusion of participants.

NHANES is an ongoing national survey project managed by the National Center for Health Statistics (NCHS), aimed at assessing the health and nutritional status of American adults and children. Participant selection follows a stratified, multistage sampling design utilized by NHANES. All participants provided written informed consent before participating in the study. Because glaucoma‐related variables were only available in the 2005–2008 NHANES cycles, the present analysis was restricted to data from 2005–2006 and 2007–2008 that simultaneously included information on both glaucoma and stroke, totaling 20,497 samples. After filtering for participants over the age of 18, 11,335 remained. Samples with uncertain stroke status were further reduced, leaving 10,887. Those with uncertain glaucoma status were also excluded, resulting in 7016 samples. After removing samples missing other covariates, 1312 remained. Figure 1 illustrates the process of participant inclusion and exclusion.

#### 2.1.2. Definition of Diseases and Related Covariates

The diagnosis of stroke, glaucoma, and diabetes was ascertained using standardized health condition questionnaires from the NHANES protocol. Specifically, disease status was based on self‐reported physician diagnosis, using the NHANES questionnaire items for stroke (MCQ160F), glaucoma (VIQ090), and diabetes (DIQ010). These operational definitions are consistent with those employed in numerous prior epidemiological studies using NHANES data (https://wwwn.cdc.gov/nchs/nhanes/analyticguidelines.aspx). Cardiovascular disease is defined based on any reported chest pain or discomfort; racial categories include Mexican American, non‐Hispanic Black, non‐Hispanic White, other Hispanic, and other race; education levels are classified as 9th grade, college graduate or above, high school grad/GED or equivalent, less than 9th grade, and some college or AA degree; marital status is identified as divorced, living with partner, married, never married, separated, and widowed; smoking status is defined as the current smoking situation, including every day, not at all, and some days; drinking criteria are based on whether at least 12 drinks of any type of alcoholic beverage were consumed in any one year. Clinical and laboratory measures, such as blood pressure, body mass index, and lipid profiles, were based on physical examinations and laboratory tests following the NHANES protocol. The complete list of all variables, their corresponding NHANES codes, and detailed descriptions are provided in Supporting Table 1. We acknowledge that the use of self‐reported data for disease status may introduce nondifferential misclassification bias, which could attenuate observed associations toward the null; this limitation is considered in the interpretation of our findings.

#### 2.1.3. Statistical Analysis

All statistical analyses were conducted using *R* (Version 4.4.1). Logistic regression was employed to assess associations. Covariates were selected a priori based on two principal criteria: (1) established evidence from previous epidemiological studies and clinical guidelines identifying the most stable and commonly adjusted major risk factors for stroke and (2) the availability of consistently measured data in the NHANES 2005–2008 cycles. The crude model includes only glaucoma as a variable. Model 1 was adjusted for basic sociodemographic factors, including age, gender, race, education level, and marital status. Model 2 further adjusted for well‐recognized cardiovascular and metabolic risk factors for stroke, including hypertension, hyperlipidemia, diabetes, cardiovascular diseases, BMI, smoking status, and alcohol consumption, which have been consistently reported as important confounders in prior studies [13–15].

### 2.2. MR Analysis

#### 2.2.1. Study Design

**FIGURE 2:**
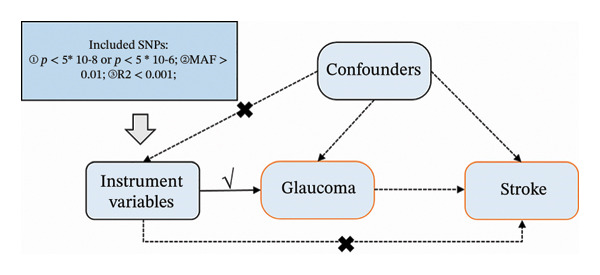
Study design and workflow.

Our approach involves a two‐sample MR design, adhering to the MR‐STROBE guidelines [16] (Figure 2). We examine causal relationships between subtypes of glaucoma—glaucoma (multitrait analysis, a genetic study combining information across related traits), glaucoma (primary open‐angle), and neovascular glaucoma—as exposure factors and various stroke subtypes—stroke, ischemic stroke (large artery atherosclerosis), ischemic stroke, ischemic stroke (small‐vessel), and ischemic stroke (cardioembolic)—as outcome variables. Our MR analysis is anchored in three foundational assumptions [17]: First, the genetic variants used as IVs must be strongly associated with the exposure; second, these variants should not be associated with any confounders of the exposure–outcome relationship; and third, the genetic variants affect the outcome solely through their effect on the exposure.

#### 2.2.2. Data Sources

We sourced GWAS summary data from published literature for glaucoma [18] and glaucoma (primary open‐angle) [19], encompassing 133,492 and 15,229 European patients, respectively, with control groups of 90,939 and 177,473. Neovascular glaucoma data were obtained from the FinnGen database, involving 1203 European patients and 400,157 controls. Additionally, data for stroke, ischemic stroke (large artery atherosclerosis), ischemic stroke, ischemic stroke (small‐vessel), and ischemic stroke (cardioembolic) are from the literature, with case/control totals of 40,585/406,111, 4373/406,111, 34,217/406,111, 5386/192,662, and 7193/211,763, respectively. For further details, see Supporting Table 1.

#### 2.2.3. Instrumental Variable Selection

The selection of IVs in this study adheres to stringent criteria to ensure robustness: Initially, SNPs must be significantly associated with glaucoma at a genome‐wide level, specifically requiring a *p* < 5 × 10^−8^. For neovascular glaucoma, due to the limited number of SNPs, this criterion is relaxed to *p* < 5 × 10^−6^ [20]. SNPs must have a minor allele frequency (MAF) greater than 0.01. To mitigate linkage disequilibrium (LD) effects (the nonrandom association of genetic variants close to each other on a chromosome), SNPs are selected based on an *R* ^2^ < 0.001 within a window size of 10,000 kb [21]. If an IV is absent in the outcome’s summary data, a SNP with high LD (*R* ^2^ > 0.8) is used as a proxy [22]. The strength of each SNP as an IV is assessed by calculating the F‐statistic, using the following formula: *F* = *R* ^2^ ∗ (N‐2)/(1‐*R* ^2^), with an *F* statistic > 10 required to avoid weak instrument bias [23].

#### 2.2.4. MR Analysis

The primary methodology for this MR study is the inverse‐variance weighted (IVW) method, which calculates the OR and its 95% CI to evaluate the causal effect. IVW was utilized as the primary estimator; when Cochran’s *Q* suggested heterogeneity, we applied a random‐effects IVW model to obtain more conservative estimates. To ensure the robustness of our findings, additional methods including MR‐Egger, weighted median, and weighted mode are utilized as complementary methods. All analyses are conducted using *R* (Version 4.3.2) with the “TwoSampleMR” package [24]. Results are illustrated through forest plots, scatter plots, and funnel plots, with a *p* < 0.05 deemed statistically significant.

#### 2.2.5. Sensitivity Analysis

In MR studies, sensitivity analysis is crucial for identifying and addressing possible pleiotropy issues. This study employs Cochran’s *Q* test to evaluate the heterogeneity among IVs [25]; a nonsignificant *p* value (greater than 0.05) suggests that heterogeneity is insignificant, implying that the variability in estimates among IVs is random and does not notably affect the IVW results. Specifically, Cochran’s *Q* was used to assess between‐instrument heterogeneity. The MR‐Egger regression method assesses the potential impact of genetic variation pleiotropy (where a single genetic variant influences multiple traits) on the results [26]; an intercept close to zero and statistically insignificant indicates no substantial pleiotropy bias. The MR‐Egger intercept was used to evaluate directional (horizontal) pleiotropy. Furthermore, the MR pleiotropy residual and outlier test (MR‐PRESSO) is applied to detect and exclude potential outliers, specifically SNPs with a *p* value less than 0.05, with the causal relationship recalculated postexclusion to address possible pleiotropy biases [27]. MR‐PRESSO was used to identify potential outlier variants and reestimate causal effects after outlier correction to mitigate pleiotropy‐driven distortion. Leave‐one‐out analysis is implemented to ensure the robustness and consistency of the findings. Leave‐one‐out analyses were used to assess whether any single SNP disproportionately drove the overall causal estimates.

#### 2.2.6. Statistical Power Consideration

To assess the robustness of our null findings and estimate the minimum detectable effect size, we performed a post hoc statistical power analysis for the primary IVW MR estimates. The calculation was conducted using the online mRnd tool (https://shiny.cnsgenomics.com/mRnd/), which requires input parameters including the variance in the exposure explained by the instrumental variables (*R* ^2^), the sample size of the outcome GWAS (N), the estimated OR, and the assumed significance level (*α* = 0.05) [28]. The *R* ^2^ for each set of genetic instruments was derived from the respective exposure GWAS summary statistics.

## 3. Results

### 3.1. Cross‐Sectional Study

#### 3.1.1. Participant Baseline Characteristics

This study analyzed data from the NHANES collected from 2005 to 2008, which included 1312 participants. Table 1 presents the baseline characteristics of individuals with and without stroke. Significant differences were noted in age, hypertension, diabetes, glycohemoglobin levels, coronary heart disease, angina, and heart attacks between stroke patients and controls, while no significant differences were found in gender, race, or lipid levels.

**TABLE 1:** Analysis of baseline characteristics of participants and the association between glaucoma and stroke.

**Part A: Baseline characteristics of study participants**				
	**No** ***N* = 1222**	**Yes** ***N* = 90**	**p** **.overall**	

Age	59.9 (12.2)	68.2 (11.1)	< 0.001	
Gender			0.817	
Female	484 (39.6%)	34 (37.8%)		
Male	738 (60.4%)	56 (62.2%)		
Race			0.409	
Mexican American	159 (13.0%)	8 (8.89%)		
Non‐Hispanic Black	206 (16.9%)	22 (24.4%)		
Non‐Hispanic White	746 (61.0%)	52 (57.8%)		
Other Hispanic	79 (6.46%)	6 (6.67%)		
Other race—including multiracial	32 (2.62%)	2 (2.22%)		
Education			0.067	
9–11th grade (includes 12th grade with no diploma)	196 (16.0%)	17 (18.9%)		
College graduate or above	225 (18.4%)	9 (10.0%)		
High school grad/GED or equivalent	317 (25.9%)	23 (25.6%)		
Less than 9th grade	165 (13.5%)	21 (23.3%)		
Refused	1 (0.08%)	0 (0.00%)		
Some college or AA degree	318 (26.0%)	20 (22.2%)		
Marriage			0.575	
Divorced	192 (15.7%)	13 (14.4%)		
Living with partner	76 (6.22%)	3 (3.33%)		
Married	691 (56.5%)	50 (55.6%)		
Never married	79 (6.46%)	5 (5.56%)		
Separated	44 (3.60%)	3 (3.33%)		
Widowed	140 (11.5%)	16 (17.8%)		
Diastolic_blood_pressure	70.0 (13.8)	68.1 (16.9)	0.292	
Systolic_blood_pressure	128 (19.5)	134 (23.0)	0.024	
Hypertension			< 0.001	
No	702 (57.4%)	19 (21.1%)		
Yes	520 (42.6%)	71 (78.9%)		
LDL‐C	117 (37.3)	108 (43.3)	0.066	
HDL‐C	54.6 (17.0)	53.0 (19.9)	0.448	
Diabetes:			< 0.001	
Borderline	29 (2.37%)	1 (1.11%)		
No	1037 (84.9%)	60 (66.7%)		
Yes	156 (12.8%)	29 (32.2%)		
Glycohemoglobin	5.77 (0.99)	6.14 (1.25)	0.008	
Glucose_plasma	111 (31.9)	121 (47.4)	0.052	
Coronary_heart_disease			< 0.001	
No	1131 (92.6%)	71 (78.9%)		
Yes	91 (7.45%)	19 (21.1%)		
Angina			0.001	
No	1160 (94.9%)	77 (85.6%)		
Yes	62 (5.07%)	13 (14.4%)		
Heart_attack			< 0.001	
No	1125 (92.1%)	66 (73.3%)		
Yes	97 (7.94%)	24 (26.7%)		
BMI	28.4 (5.90)	29.1 (6.13)	0.331	
Glaucoma			0.644	
No	1149 (94.0%)	83 (92.2%)		
Yes	73 (5.97%)	7 (7.78%)		
Smoking			0.398	
Every day	424 (34.7%)	25 (27.8%)		
Not at all	750 (61.4%)	62 (68.9%)		
Some days	48 (3.93%)	3 (3.33%)		
Drinking			0.502	
No	242 (19.8%)	21 (23.3%)		
Yes	980 (80.2%)	69 (76.7%)		

**Part B: Association analysis between glaucoma and stroke risk (NHANES 2005–2006 + 2007–2008)**				
	**OR**	**lower_95% CI**	**upper_95% CI**	**p** **value**

Crude model	1.327447	0.541814269	2.787071209	0.491412
Model 1	0.822636	0.328518395	1.779672023	0.646011
Model 2	0.728301	0.281583183	1.635461252	0.474386

#### 3.1.2. Association Between Glaucoma and Stroke

Table 1 details the correlation analysis between glaucoma and stroke risk. The initial crude model, incorporating only glaucoma, indicated no significant association between glaucoma and stroke risk (OR = 1.327, 95% CI: 0.541–2.787, *p* = 0.491). After adjusting for age, gender, race, education level, income, and marital status, Model 1 still exhibited no significant correlation (OR = 0.822, 95% CI: 0.328–1.779, *p* = 0.646). Furthermore, Model 2, which additionally adjusted for hypertension, hyperlipidemia, diabetes, cardiovascular diseases, BMI, smoking status, and alcohol consumption, continued to show no significant association (OR = 0.728, 95% CI: 0.281–1.635, *p* = 0.474). Given the reduced analytic sample after excluding participants with missing covariates, these estimates may be underpowered to detect modest associations.

### 3.2. MR Analysis

#### 3.2.1. Selection of IVs

For this study, various genetic markers linked to glaucoma were selected as IVs for the MR analysis. In the MR analysis of glaucoma (multi‐trait analysis), 98 IVs were identified. Among these, 2 SNPs (rs2439042 and rs722585) lacked matching data in the summary datasets, hence proxies rs2253597 and rs3800056 were utilized, respectively. In analyses involving ischemic stroke (large artery atherosclerosis) and other subtypes, the same SNP (rs2439042) required replacement, with rs2253597 serving as the proxy. For glaucoma (primary open‐angle), 46 IVs were chosen; in cases where stroke was the outcome, 2 SNPs (rs1649068 and rs2735114) lacked requisite data and were substituted by rs1649067 and rs1059509, respectively. In scenarios examining ischemic stroke (cardioembolic) outcomes, rs2735114 was substituted with rs1059509. For neovascular glaucoma, 17 pertinent IVs were selected, with one (rs183574534) missing necessary data. The F‐statistics for all selected IVs exceeded 10, indicating robust statistical power (Supporting Table 2).

#### 3.2.2. MR Analysis Results

Despite previous clinical reports suggesting associations, IVW showed no evidence of a causal association between genetically predicted glaucoma and stroke, including glaucoma (multitrait analysis) (OR: 1.01, 95% CI: 0.99–1.04, *p* = 0.28), glaucoma (primary open‐angle) (OR: 1.01, 95% CI: 0.98–1.03, *p* = 0.65), and neovascular glaucoma (OR: 1.01, 95%CI: 0.99–1.03, *p* = 0.41). No statistically significant causal associations were observed between any glaucoma subtype and any ischemic stroke subtype. Consistently, MR‐Egger, weighted median, and weighted mode analyses yielded directionally similar and nonsignificant estimates (Table 2), supporting the robustness of the null findings across MR estimators. Visual representations in scatter plots and forest plots further confirm the uniformity of these findings (Supporting Figure 1).

**TABLE 2:** Mendelian randomization analysis of causal relationships between glaucoma and stroke.

**Part A: Primary causal analysis results (IVW method)**								
**Exposure**	**Outcome**	**Significant of SNP**	**N.SNPs**	**Methods**	**OR (95% CI)**	**p**		

Glaucoma (primary open‐angle)	Stroke	5 × 10^−8^	43	IVW	1.01 (0.98–1.03)	0.65		
Glaucoma (primary open‐angle)	Stroke		43	MR‐Egger	0.98 (0.90–1.06)	0.59		
Glaucoma (primary open‐angle)	Stroke		43	Weighted median	1.01 (0.97–1.05)	0.63		
Glaucoma (primary open‐angle)	Stroke		43	Weighted mode	1.01 (0.94–1.09)	0.73		
Glaucoma (primary open‐angle)	Ischemic stroke (large artery atherosclerosis)	5 × 10^−8^	43	IVW	1.04 (0.97–1.13)	0.26		
Glaucoma (primary open‐angle)	Ischemic stroke (large artery atherosclerosis)		43	MR‐Egger	1.10 (0.86–1.40)	0.45		
Glaucoma (primary open‐angle)	Ischemic stroke (large artery atherosclerosis)		43	Weighted median	1.09 (0.98–1.22)	0.11		
Glaucoma (primary open‐angle)	Ischemic stroke (large artery atherosclerosis)		43	Weighted mode	1.12 (0.94–1.34)	0.22		
Glaucoma (primary open‐angle)	Ischemic stroke	5 × 10^−8^	43	IVW	1.01 (0.98–1.04)	0.47		
Glaucoma (primary open‐angle)	Ischemic stroke		43	MR‐Egger	0.98 (0.89–1.08)	0.67		
Glaucoma (primary open‐angle)	Ischemic stroke		43	Weighted median	1.00 (0.95–1.04)	0.86		
Glaucoma (primary open‐angle)	Ischemic stroke		43	Weighted mode	0.98 (0.91–1.05)	0.60		
Glaucoma (primary open‐angle)	Ischemic stroke (small‐vessel)	5 × 10^−8^	45	IVW	1.02 (0.96–1.09)	0.52		
Glaucoma (primary open‐angle)	Ischemic stroke (small‐vessel)		45	MR‐Egger	1.05 (0.87–1.27)	0.61		
Glaucoma (primary open‐angle)	Ischemic stroke (small‐vessel)		45	Weighted median	1.09 (0.99–1.21)	0.09		
Glaucoma (primary open‐angle)	Ischemic stroke (small‐vessel)		45	Weighted mode	1.17 (0.99–1.37)	0.07		
Glaucoma (primary open‐angle)	Ischemic stroke (cardioembolic)	5 × 10^−8^	45	IVW	0.96 (0.91–1.01)	0.13		
Glaucoma (primary open‐angle)	Ischemic stroke (cardioembolic)		45	MR‐Egger	0.92 (0.78–1.07)	0.28		
Glaucoma (primary open‐angle)	Ischemic stroke (cardioembolic)		45	Weighted median	0.97 (0.90–1.05)	0.48		
Glaucoma (primary open‐angle)	Ischemic stroke (cardioembolic)		45	Weighted mode	0.95 (0.87–1.05)	0.33		
Glaucoma (multitrait analysis)	Stroke	5 × 10^−8^	92	IVW	1.01 (0.99–1.04)	0.28		
Glaucoma (multitrait analysis)	Stroke		92	MR‐Egger	1.02 (0.94–1.09)	0.69		
Glaucoma (multitrait analysis)	Stroke		92	Weighted median	1.01 (0.97–1.05)	0.56		
Glaucoma (multitrait analysis)	Stroke		92	Weighted mode	1.00 (0.94–1.07)	0.97		
Glaucoma (multitrait analysis)	Ischemic stroke (large artery atherosclerosis)	5 × 10^−8^	93	IVW	1.00 (0.93–1.08)	1.00		
Glaucoma (multitrait analysis)	Ischemic stroke (large artery atherosclerosis)		93	MR‐Egger	1.20 (0.98–1.47)	0.08		
Glaucoma (multitrait analysis)	Ischemic stroke (large artery atherosclerosis)		93	Weighted median	1.08 (0.97–1.19)	0.15		
Glaucoma (multi‐trait analysis)	Ischemic stroke (large artery atherosclerosis)		93	Weighted mode	1.13 (0.97–1.33)	0.13		
Glaucoma (multitrait analysis)	Ischemic stroke	5 × 10^−8^	93	IVW	1.01 (0.98–1.04)	0.35		
Glaucoma (multitrait analysis)	Ischemic stroke		93	MR‐Egger	1.00 (0.92–1.08)	0.95		
Glaucoma (multitrait analysis)	Ischemic stroke		93	Weighted median	1.01 (0.97–1.06)	0.48		
Glaucoma (multitrait analysis)	Ischemic stroke		93	Weighted mode	1.00 (0.92–1.08)	0.91		
Glaucoma (multitrait analysis)	Ischemic stroke (small‐vessel)	5 × 10^−8^	94	IVW	1.00 (0.94–1.06)	0.91		
Glaucoma (multitrait analysis)	Ischemic stroke (small‐vessel)		94	MR‐Egger	0.99 (0.83–1.19)	0.95		
Glaucoma (multitrait analysis)	Ischemic stroke (small‐vessel)		94	Weighted median	1.02 (0.93–1.11)	0.75		
Glaucoma (multitrait analysis)	Ischemic stroke (small‐vessel)		94	Weighted mode	1.01 (0.84–1.20)	0.95		
Glaucoma (multitrait analysis)	Ischemic stroke (cardioembolic)	5 × 10^−8^	94	IVW	0.96 (0.91–1.01)	0.14		
Glaucoma (multitrait analysis)	Ischemic stroke (cardioembolic)		94	MR‐Egger	0.96 (0.83–1.11)	0.55		
Glaucoma (multitrait analysis)	Ischemic stroke (cardioembolic)		94	Weighted median	0.98 (0.91–1.06)	0.68		
Glaucoma (multitrait analysis)	Ischemic stroke (cardioembolic)		94	Weighted mode	0.99 (0.87–1.12)	0.82		
Neovascular glaucoma	Stroke	5 × 10^−6^	16	IVW	1.01 (0.99–1.03)	0.41		
Neovascular glaucoma	Stroke		16	MR‐Egger	1.03 (0.98–1.08)	0.26		
Neovascular glaucoma	Stroke		16	Weighted median	1.01 (0.98–1.04)	0.67		
Neovascular glaucoma	Stroke		16	Weighted mode	1.00 (0.97–1.04)	0.81		
Neovascular glaucoma	Ischemic stroke (large artery atherosclerosis)	5 × 10^−6^	16	IVW	1.04 (0.98–1.10)	0.25		
Neovascular glaucoma	Ischemic stroke (large artery atherosclerosis)		16	MR‐Egger	1.09 (0.95–1.25)	0.22		
Neovascular glaucoma	Ischemic stroke (large artery atherosclerosis)		16	Weighted median	1.02 (0.94–1.11)	0.67		
Neovascular glaucoma	Ischemic stroke (large artery atherosclerosis)		16	Weighted mode	1.00 (0.89–1.14)	0.95		
Neovascular glaucoma	Ischemic stroke	5 × 10^−6^	16	IVW	1.01 (0.98–1.03)	0.49		
Neovascular glaucoma	Ischemic stroke		16	MR‐Egger	1.01 (0.96–1.07)	0.70		
Neovascular glaucoma	Ischemic stroke		16	Weighted median	1.01 (0.97–1.04)	0.74		
Neovascular glaucoma	Ischemic stroke		16	Weighted mode	1.00 (0.96–1.05)	0.86		
Neovascular glaucoma	Ischemic stroke (small‐vessel)	5 × 10^−6^	16	IVW	1.02 (0.96–1.08)	0.49		
Neovascular glaucoma	Ischemic stroke (small‐vessel)		16	MR‐Egger	1.09 (0.96–1.23)	0.21		
Neovascular glaucoma	Ischemic stroke (small‐vessel)		16	Weighted median	1.00 (0.93–1.07)	0.93		
Neovascular glaucoma	Ischemic stroke (small‐vessel)		16	Weighted mode	1.00 (0.90–1.11)	0.99		
Neovascular glaucoma	Ischemic stroke (cardioembolic)	5 × 10^−6^	16	IVW	1.01 (0.96–1.06)	0.81		
Neovascular glaucoma	Ischemic stroke (cardioembolic)		16	MR‐Egger	1.05 (0.93–1.17)	0.46		
Neovascular glaucoma	Ischemic stroke (cardioembolic)		16	Weighted median	1.02 (0.95–1.09)	0.62		
Neovascular glaucoma	Ischemic stroke (cardioembolic)		16	Weighted mode	1.08 (0.93–1.27)	0.32		

**Part B: Heterogeneity and pleiotropy assessment**								
**Exposure**	**Outcome**	**Heterogeneity**			**Pleiotropy**			
		**Q statistic (IVW)**	**p** **value**	**FDR adjusted** **p** **value**	**MR-Egger intercept**	**p** **value**	**FDR adjusted** **p** **value**	

Glaucoma (primary open‐angle)	Stroke	93.19	< 0.001	< 0.001	−0.0091	0.176	0.698	
Glaucoma (primary open‐angle)	Ischemic stroke (large artery atherosclerosis)	73.28	0.004	0.009	−0.0331	0.035	0.265	
Glaucoma (primary open‐angle)	Ischemic stroke	105.69	< 0.001	< 0.001	−0.0102	0.186	0.698	
Glaucoma (primary open‐angle)	Ischemic stroke (small‐vessel)	50.89	0.221	0.348	−0.0038	0.759	0.949	
Glaucoma (primary open‐angle)	Ischemic stroke (cardioembolic)	50.51	0.232	0.348	0.0061	0.552	0.828	
Glaucoma (multitrait analysis)	Stroke	170.46	< 0.001	< 0.001	−0.0030	0.487	0.811	
Glaucoma (multitrait analysis)	Ischemic stroke (large artery atherosclerosis)	145.55	< 0.001	0.001	−0.0234	0.026	0.265	
Glaucoma (multitrait analysis)	Ischemic stroke	182.49	< 0.001	< 0.001	−0.0020	0.677	0.923	
Glaucoma (multitrait analysis)	Ischemic stroke (small‐vessel)	111.81	0.089	0.192	0.0002	0.979	0.979	
Glaucoma (multi‐trait analysis)	Ischemic stroke (cardioembolic)	105.99	0.169	0.316	0.0005	0.939	0.979	
Neovascular glaucoma	Stroke	11.62	0.708	0.758	−0.0076	0.388	0.811	
Neovascular glaucoma	Ischemic stroke (large artery atherosclerosis)	14.59	0.481	0.601	−0.0201	0.402	0.811	
Neovascular glaucoma	Ischemic stroke	12.42	0.647	0.747	−0.0009	0.925	0.979	
Neovascular glaucoma	Ischemic stroke (small‐vessel)	8.40	0.907	0.907	−0.0242	0.275	0.811	
Neovascular glaucoma	Ischemic stroke (cardioembolic)	16.88	0.326	0.445	−0.0141	0.479	0.811	

**Part C: MR-PRESSO outlier detection and correction**								
**Exposure**	**Outcome**	**Raw**		**Outlier corrected**		**Global** *p*	**Number of outliers**	**Distortion** **p**
		**OR (CI%)**	**p**	**OR (CI%)**	**p**			

Glaucoma (multitrait analysis)	Stroke	1.00 (0.97–1.04)	0.802	1.01 (0.99–1.04)	0.283	< 0.001	2 (rs3184504,rs4102217	0.680
Neovascular glaucoma	Stroke	1.01 (0.99–1.03)	0.363	NA (NA ‐NA)	NA	0.743	NA	NA
Glaucoma (primary open‐angle)	Stroke	1.01 (0.98–1.05)	0.541	1.01 (0.98–1.03)	0.625	< 0.001	2 (rs6475604,rs7137828)	0.335
Glaucoma (multitrait analysis)	Ischemic stroke (large artery atherosclerosis)	0.99 (0.92–1.07)	0.799	1.00 (0.93–1.08)	0.996	< 0.001	1 (rs3184504)	0.008
Glaucoma (primary open‐angle)	Ischemic stroke (large artery atherosclerosis)	1.08 (0.99–1.18)	0.094	1.04 (0.97–1.13)	0.271	0.003	2 (rs6475604,rs7137828)	0.131
Neovascular glaucoma	Ischemic stroke (large artery atherosclerosis)	1.04 (0.98–1.10)	0.260	NA (NA ‐ NA)	NA	0.456	NA	NA
Glaucoma (multitrait analysis)	Ischemic stroke	1.01 (0.97–1.04)	0.735	1.01 (0.98–1.04)	0.354	< 0.001	1 (rs3184504)	0.744
Glaucoma (primary open‐angle)	Ischemic stroke	1.02 (0.97–1.06)	0.483	1.01 (0.98–1.04)	0.470	< 0.001	2 (rs6475604,rs7137828)	0.579
Neovascular glaucoma	Ischemic stroke	1.01 (0.99–1.03)	0.464	NA (NA ‐ NA)	NA	0.682	NA	NA
Glaucoma (primary open‐angle)	Ischemic stroke (small‐vessel)	1.02 (0.96–1.09)	0.526	NA (NA ‐ NA)	NA	0.212	NA	NA
Glaucoma (multitrait analysis)	Ischemic stroke (small‐vessel)	1.00 (0.94–1.06)	0.908	NA (NA ‐ NA)	NA	0.078	NA	NA
Neovascular glaucoma	Ischemic stroke (small‐vessel)	1.02 (0.98–1.06)	0.373	NA (NA ‐ NA)	NA	0.927	NA	NA
Glaucoma (multitrait analysis)	Ischemic stroke (cardioembolic)	0.96 (0.91–1.01)	0.144	NA (NA ‐ NA)	NA	0.158	NA	NA
Glaucoma (primary open‐angle)	Ischemic stroke (cardioembolic)	0.96 (0.91–1.01)	0.138	NA (NA ‐ NA)	NA	0.244	NA	NA
Neovascular glaucoma	Ischemic stroke (cardioembolic)	1.01 (0.96–1.06)	0.812	NA (NA ‐ NA)	NA	0.299	NA	NA

#### 3.2.3. Sensitivity Analysis

Heterogeneity was first assessed using Cochran’s *Q* test. We observed significant heterogeneity for primary open‐angle glaucoma with stroke, ischemic stroke (large artery atherosclerosis), and ischemic stroke (*p* < 0.001, 0.004, and < 0.001, respectively), and similarly for glaucoma (multitrait analysis) with stroke, ischemic stroke (large artery atherosclerosis), and ischemic stroke (all *p* < 0.001). Accordingly, IVW estimates were obtained using a random‐effects model in these analyses to account for between‐instrument heterogeneity. Funnel plot symmetry provided additional support for the absence of major small‐study effects or directional distortion, suggesting that the observed heterogeneity did not materially influence the overall conclusions (Supporting Figure 2).

Directional horizontal pleiotropy (where genetic variants influence the outcome through pathways independent of the exposure) was then evaluated using the MR‐Egger intercept. Evidence suggestive of horizontal pleiotropy was observed for the outcome ischemic stroke (large artery atherosclerosis) when using primary open‐angle glaucoma and glaucoma (multitrait analysis) as exposures (*p* = 0.035 and 0.026, respectively; Table 2).

Next, MR‐PRESSO was used to identify outlier instruments potentially driving pleiotropic distortion. For stroke as the outcome, MR‐PRESSO detected two outliers when using glaucoma (multitrait analysis) and glaucoma (primary open‐angle) as exposures (rs3184504 and rs4102217). When glaucoma (primary open‐angle) was the exposure, two outliers were identified in analyses for stroke, ischemic stroke (large artery atherosclerosis), and ischemic stroke (rs6475604 and rs7137828). When glaucoma (multitrait analysis) was the exposure, one outlier was detected for ischemic stroke (large artery atherosclerosis) and ischemic stroke (rs3184504). After excluding these outliers, causal estimates remained nonsignificant (Table 2), indicating that the null associations were robust to outlier correction.

Finally, leave‐one‐out analyses did not identify any single SNP that disproportionately drove the IVW estimates (Supporting Figure 2), further supporting the stability of our results.

#### 3.2.4. Statistical Power of MR Analyses

Given the null findings from the MR analyses, we evaluated the statistical power to detect a causal effect. As detailed in Supporting Table 4, the estimated statistical power across all exposure–outcome pairs was generally low, ranging from approximately 5%–10%. This limited power is primarily attributable to the modest proportion of variance explained (*R* ^2^) by the genetic instruments for glaucoma and the observed ORs being very close to the null value of one.

## 4. Discussion

This study employed MR to examine potential causal relationships between various types of glaucoma (including multitrait, primary open‐angle, and neovascular) and ischemic stroke subtypes. Additionally, we analyzed data from the NHANES from 2005 to 2008 to assess the association between glaucoma and stroke. Our comprehensive analysis, combining MR and NHANES data, revealed no significant causal or epidemiological correlation between these glaucoma types and any stroke subtype. Although prior clinical studies suggested a higher stroke risk among glaucoma patients, our rigorous methodological approach provides more definitive evidence that genetically, glaucoma does not increase stroke risk.

A structured reevaluation of prior observational literature, organized by methodological rigor and phenotype, clarifies the discrepancy with our null findings. Early observational studies that did not sufficiently adjust for key confounders often reported strong associations. For instance, a study of 11,959 participants found that primary open‐angle glaucoma prevalence was significantly higher in stroke patients than controls (8.5% vs. 3.8%; *p* < 0.001), though this became nonsignificant after risk factor adjustment. Similarly, a Chinese study with 17,713 subjects showed a significant correlation between glaucoma and increased stroke risk (OR 5.278, 95% CI: 1.094–25.462) [8]. However, studies that implemented more comprehensive adjustment for shared vascular confounders such as hypertension and diabetes typically observed attenuated effect sizes, underscoring the critical role of residual confounding. A longitudinal study of 1,025,340 subjects found that the initially elevated stroke likelihood among individuals with open‐angle glaucoma (HR = 1.2, 95% CI: 1.03–1.40) was substantially influenced by these shared risk factors [29]. A meta‐analysis of seven studies involving 362,267 participants confirmed an association between glaucoma and increased stroke risk (OR = 1.94, 95% CI = 1.45–2.59) but also highlighted substantial between‐study heterogeneity, largely attributable to differing levels of confounder control [6]. Furthermore, observations varied across specific glaucoma subtypes. For neovascular glaucoma, research indicates 15.6% higher cumulative ischemic stroke incidence compared to controls, with a risk ratio of 2.07 (95% CI: 1.41–3.02). For a normal tension glaucoma (NTG), a clinical study of 1218 patients reported a significantly higher stroke incidence (hazard ratio HR = 6.34) [30]. Additionally, a study separating adolescent patients with open‐angle glaucoma into high and low intraocular pressure groups found increased stroke risks in both, with the high‐pressure group exhibiting a higher risk ratio (OR, 2.58; 95% CI, 1.20–5.53) compared to the low‐pressure group (OR, 1.91; 95% CI, 1.12–3.25) [31]. This heterogeneity suggests that distinct pathophysiologies or comorbid profiles, rather than glaucoma per se, may drive subtype‐specific risks reported in some settings.

Our MR study, by using genetic instruments to minimize confounding and by evaluating specific glaucoma subtypes, addresses these core methodological limitations. The consistent null findings across our analyses suggest that the positive signals from observational studies are more likely attributable to shared risk factors, residual confounding, or diagnostic bias than to a direct causal effect of glaucoma on stroke.

Although our study did not identify a significant causal association between glaucoma and stroke, this does not negate the possibility that glaucoma might influence stroke risk through specific biological pathways. Contemporary research proposes several mechanisms through which glaucoma could impact stroke risk, including alterations in cerebral vascular function and blood flow influenced by intraocular pressure, as well as shared risk factors such as hypertension and neurological dysfunction. Studies show that glaucoma patients exhibit reduced cerebral artery blood flow velocity, potentially impairing vascular reactivity [32], and a significant correlation between stroke and open‐angle glaucoma in hypertensive patients (OR = 2.059). Additionally, glaucoma may induce autonomic nervous system dysfunction, further predisposing individuals to stroke. Beyond hemodynamics, immune and inflammatory mechanisms are increasingly implicated across neurovascular diseases, providing a plausible shared substrate for ocular and cerebral vulnerability; therefore, integrating vascular imaging with immune‐phenotyping and multiomics may help disentangle shared pathways in future work [12].

Our sensitivity analyses support the robustness of the null findings. Heterogeneity was observed in several analyses, and we therefore applied random‐effects IVW where appropriate. Although MR‐Egger suggested possible directional pleiotropy in specific models, MR‐PRESSO and leave‐one‐out analyses did not materially change the estimates, while acknowledging that residual pleiotropy or phenotype definition differences cannot be fully excluded.

The absence of a detectable causal relationship in our MR study might stem from several issues. Firstly, the genetic variants used might have an insufficient effect size to reveal a significant impact of glaucoma on stroke risk. Secondly, the types of glaucoma represented in the sample may not cover all subtypes extensively, possibly overlooking some that are more significantly associated with stroke risk. Therefore, our findings indicate a need for further research to elucidate the direct connections and underlying mechanisms between these diseases, potentially involving more comprehensive sampling and exploration of additional genetic variants.

The primary strength lies in integrating MR with observational NHANES data, significantly reducing confounding factors and enhancing causal inference reliability. However, notable limitations exist. First, GWAS data predominantly originate from European populations. We explicitly acknowledge that this limits the generalizability of our MR findings and causal conclusions to other ethnic groups. Our results may not generalize to non‐European populations, such as African or East Asian cohorts, given that glaucoma progresses more severely in African populations [33]. Large‐scale POAG genetics in African ancestry populations is expanding and may reveal ancestry‐specific loci and effect architectures, underscoring the importance of multiancestry replication for external validity [34]. Second, the cross‐sectional NHANES design may not adequately capture causal relationships and contain potential selection biases. The substantial reduction in the NHANES analytic sample (from 20,497 to 1312) occurred primarily due to missing covariate data, which may introduce selection bias and affect the external validity of the observational estimates if participants with complete data differ systematically from those excluded. Third, the disease definitions used may not fully represent the potential glaucoma–stroke relationship. Furthermore, the reliance on self‐reported physician diagnoses for both glaucoma and stroke in NHANES may lead to misclassification. Such misclassification is likely nondifferential, which would bias the observed association toward the null and could partially explain the null findings in our cross‐sectional analysis. Additionally, although we adjusted for major established confounders based on clinical guidelines and data availability, not all potential stroke risk factors, such as sleep apnea, were systematically collected in the NHANES cycles used. Incorporating variables with incomplete data would have necessitated the exclusion of additional participants, thereby further reducing the analytic sample size and statistical power. This represents a common constraint in secondary analyses of publicly available cohort data. Finally, while this study identifies evidence of causal relationships, it does not explore underlying biological mechanisms in detail. Future research should employ larger sample sizes and advanced methodologies to validate findings and explore biological pathways. We restricted the NHANES analysis to 2005–2008 because these were the only cycles in which both glaucoma and stroke variables (with comparable definitions) were concurrently available, which reduced the analytic sample size and may have limited statistical power, increased the risk of selection bias, and precluded assessment of the temporal sequence between glaucoma and stroke. In addition, for neovascular glaucoma, the relaxed SNP selection threshold may increase susceptibility to weak instrument or false‐positive bias despite all instruments having *F*‐statistics > 10, and residual horizontal pleiotropy or phenotype misclassification cannot be completely ruled out. More fundamentally, the core MR assumption of no horizontal pleiotropy is challenging to fully verify. Glaucoma and stroke share several biological pathways, including those involved in vascular regulation and systemic metabolism. Genetic variants influencing glaucoma might affect stroke risk through these shared pathways rather than exclusively through glaucoma itself, representing a potential source of residual confounding that sensitivity analyses may not entirely eliminate. This is an inherent limitation of the MR approach.

The significant heterogeneity observed in several MR analyses may also reflect underlying phenotypic diversity. Specifically, the genetic instruments for glaucoma aggregate effects across distinct subtypes such as primary open‐angle and neovascular glaucoma, which have different pathophysiologies. Similarly, the stroke outcome combines etiologically distinct subtypes. This biological heterogeneity within both exposure and outcome is a plausible explanation for the statistical heterogeneity detected. Additionally, our MR analyses had limited statistical power (approximately 5%–10%) to detect causal effects, primarily due to the modest variance explained by the genetic instruments for glaucoma. This warrants caution in interpreting the null findings.

Given these limitations, it is imperative that future studies include more diverse samples from various ethnicities and regions to enhance the generalizability of the results. Additionally, exploring more potential biological mechanisms could elucidate specific pathways through which glaucoma may influence stroke risk.

## 5. Conclusion

In conclusion, this integrated analysis found no genetic or consistent epidemiological evidence supporting a causal relationship between glaucoma and stroke. Importantly, the absence of a direct causal link does not negate their clinical association, which is likely driven by shared vascular risk factors. Therefore, aggressive management of these common risk factors, such as hypertension and diabetes, remains essential in the clinical care of patients with either condition. By integrating data from cohort studies and MR analyses, our research consistently showed negative results, challenging prevailing research hypotheses. This underscores the necessity for future studies to devise more targeted prevention and treatment strategies. Such studies, particularly those employing longitudinal designs to further explore this relationship, are crucial for advancing our understanding of these diseases and potentially improving outcomes for patients worldwide.

## Author Contributions

Likai Shi and Yulong Ma contributed to the conception and design of the study. Dandan Chang, Yanan He, and Qingke Shi were responsible for data acquisition. Yulong Ma performed the statistical analysis and data interpretation. Yanan He, Qingke Shi, Dandan Chang, Yanshan Hu, Yulong Ma, Hang Guo, and Likai Shi drafted the manuscript. All authors critically revised the manuscript for important intellectual content. Yanshan Hu supervised administrative, technical, and material support. Likai Shi and Hang Guo obtained funding and provided research group leadership.

## Funding

The authors have nothing to report.

## Disclosure

All authors have read and approved the final version of the manuscript and agree to be accountable for all aspects of the work.

## Ethics Statement

Not applicable because NHANES and FinnGen belong to public databases, the patients involved in the database have obtained ethical approval, users can download relevant data for free for research and publish relevant articles, and our study is based on open‐source data; The First Medical Center of Chinese PLA General Hospital does not require research using publicly available data to be submitted for review to its ethics committee, so there are no ethical issues and other conflicts of interest.

## Consent

Not applicable because this paper did not reveal any personal information of patients.

## Conflicts of Interest

The authors declare no conflicts of interest.

## Supporting Information

Additional supporting information can be found online in the Supporting Information section.

## Supporting information


**Supporting Information 1** Supporting Figure 1. Scatter plots and forest plots of MR analysis. Scatter plots: glaucoma (multitrait analysis) on ischemic stroke (cardioembolic).

**Supporting Information 2** Supporting Figure 2. Funnel plots and leave‐one‐out analysis of MR analysis.

**Supporting Information 3** Supporting Table 1. Details of the GWAS summary data included in the Mendelian randomization.

**Supporting Information 4** Supporting Table 2. Details of the SNPs included in the Mendelian randomization.

**Supporting Information 5** Supporting Table 3 Definitions, NHANES codes, and data sources of variables included in the cross‐sectional analysis.

**Supporting Information 6** Supporting Table 4 Outcome.

## Data Availability

All data generated or analyzed during this study are included in this published article and its supporting information files.
