# Supplementary figures and images for: Absence of Association Between Glaucoma and Stroke Risk: Insights From a Cross‐Sectional Study and a Two‐Sample Mendelian Randomization Study
Source: J Ophthalmol. 2026 May 21;2026:5492641. doi: 10.1155/joph/5492641 (PMC13191766; doi:10.1155/joph/5492641)

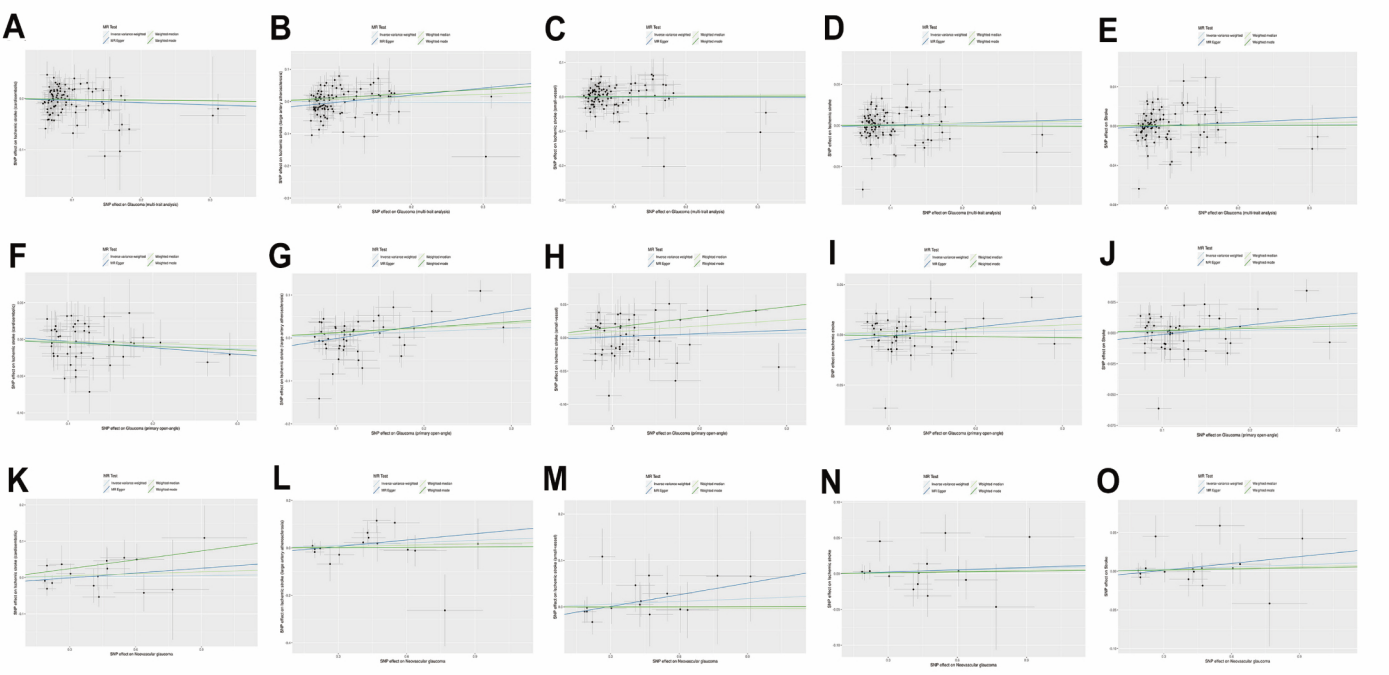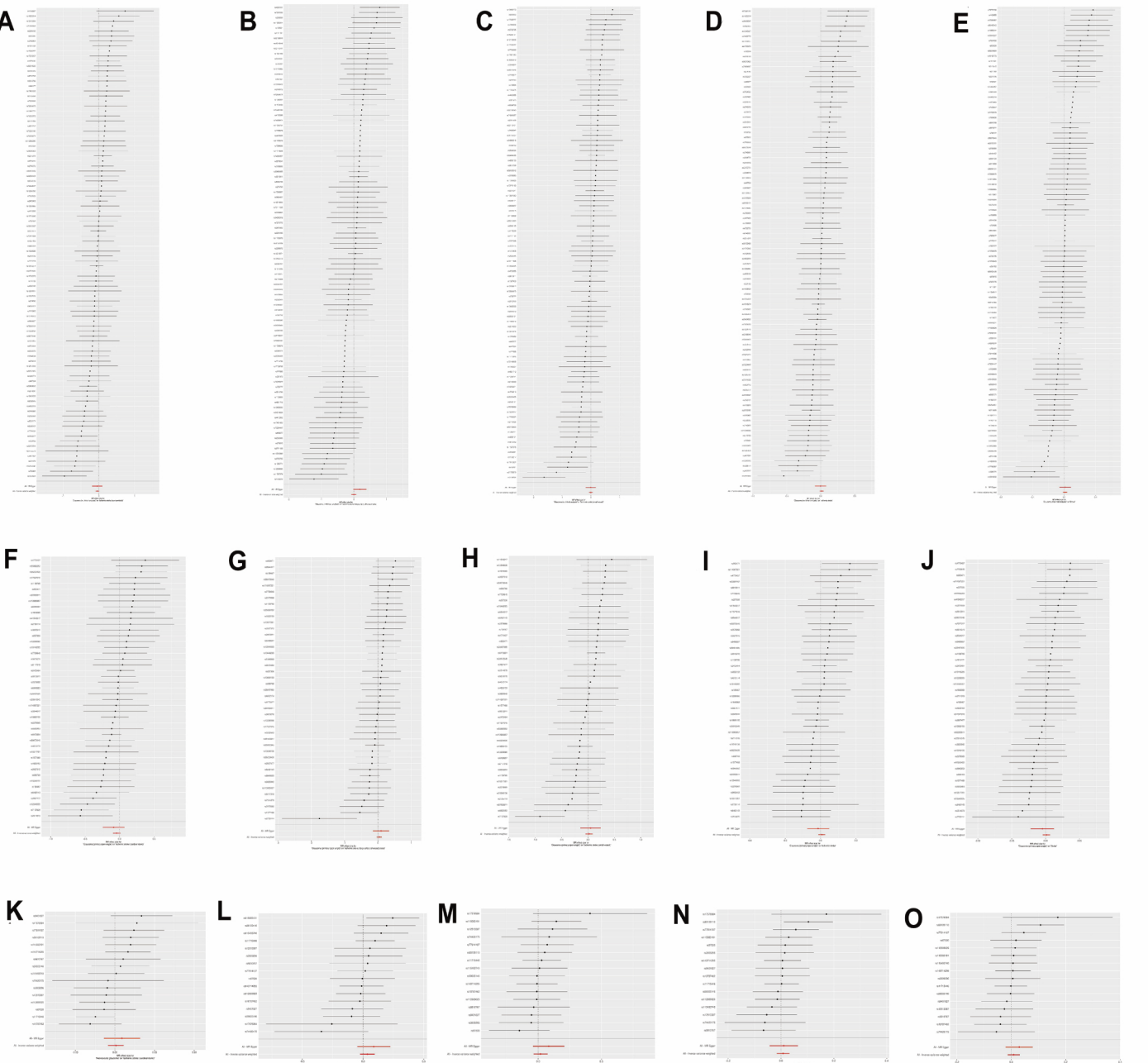

Supplement: Supplementary file 1 — Supporting Information 1 Supporting Figure 1. Scatter plots and forest plots of MR analysis. Scatter plots: glaucoma (multitrait analysis) on ischemic stroke (cardioembolic). [file JOPH-2026-5492641-s004.pdf]
