# Supplementary material for: Absence of Association Between Glaucoma and Stroke Risk: Insights From a Cross‐Sectional Study and a Two‐Sample Mendelian Randomization Study
Source: J Ophthalmol. 2026 May 21;2026:5492641. doi: 10.1155/joph/5492641 (PMC13191766; doi:10.1155/joph/5492641)
Supplement: Supplementary file 3 — Supporting Information 3 Supporting Table 1. Details of the GWAS summary data included in the Mendelian randomization. [file JOPH-2026-5492641-s003.pdf]

Supplementary Table 1. Details of the GWAS summary data included in the Mendelian randomization

Character	Traits	GWAS ID	N（case/control）	Population	Number of SNPs
Outcome	Stroke	ebi-a-GCST006906	40,585/406,111	European	8,211,693
Outcome	Ischemic stroke (large artery atherosclerosis)	ebi-a-GCST006907	4,373/406,111		8,418,349
Outcome	Ischemic stroke	ebi-a-GCST006908	34,217/406,111		8,296,492
Outcome	Ischemic stroke (small-vessel)	ebi-a-GCST006909	5,386/192,662		8,280,845
Outcome	Ischemic stroke (cardioembolic)	ebi-a-GCST006910	7,193/211,763		8,271,294
Exposure	Glaucoma (multi-trait analysis)	ebi-a-GCST009722	133,492/90,939		7,981,170
Exposure	Glaucoma (primary open-angle)	GCST90011767	15,229/ 177,473		NA
Exposure	Neovascular glaucoma	DM_NEOVASCULAR_GLAUCOMA	1,203/400,157		NA
