# Supplementary material for: Absence of Association Between Glaucoma and Stroke Risk: Insights From a Cross‐Sectional Study and a Two‐Sample Mendelian Randomization Study
Source: J Ophthalmol. 2026 May 21;2026:5492641. doi: 10.1155/joph/5492641 (PMC13191766; doi:10.1155/joph/5492641)
Supplement: Supplementary file 5 — Supporting Information 5 Supporting Table 3 Definitions, NHANES codes, and data sources of variables included in the cross‐sectional analysis. [file JOPH-2026-5492641-s002.pdf]

Supplementary Table 3 Definitions, NHANES codes, and data sources of variables included in the cross-sectional analysis.

Traits	ID	SAS Label	Variable Description
Age	RIDAGEYR	Age at Screening Adjudicated - Recode	Best age in years of the sample person at time of HH screening. Individuals 85 and over are topcoded at 85 years of age.
Gender	RIAGENDR	Gender	Gender of the sample person
Race	RIDRETH1	Race/Ethnicity - Recode	Recode of reported race and ethnicity information.
Education	DMDHREDU	HH Ref Person Education Level	What is the highest grade or level of school {you have/NON_SP HEAD has} received?
Marriage	DMDMARTL	Marital Status	Marital Status
Diastolic_blood_pressure	BPXDI1	Diastolic: Blood pres (1st rdg) mm Hg	Diastolic: Blood pressure (first reading) mm Hg
Systolic_blood_pressure	BPXSY1	Systolic: Blood pres (1st rdg) mm Hg	Systolic: Blood pressure (first reading) mm Hg
Hypertension	BPQ020	Ever told you had high blood pressure	{Have you/Has SP} ever been told by a doctor or other health professional that {you/s/he} had hypertension, also called high blood pressure?
LDL-C	LBDLDL	LDL-cholesterol (mg/dL)	LDL-cholesterol (mg/dL)
HDL-C	LBDHDD	Direct HDL-Cholesterol (mg/dL)	Direct HDL-Cholesterol (mg/dL)
Diabetes	DIQ010	Doctor told you have diabetes	The next questions are about specific medical conditions. {Other than during pregnancy, {have you/has SP}/{Have you/Has SP}} ever been told by a doctor or health professional that {you have/{he/she/SP} has} diabetes or sugar diabetes?
Glycohemoglobin	LBXGH	Glycohemoglobin (%)	Glycohemoglobin (%)
Glucose_plasma	LBXGLU	Fasting Glucose (mg/dL)	Fasting Glucose (mg/dL)
Coronary_Heart_Disease	MCQ160C	Ever told you had coronary heart disease	Has a doctor or other health professional ever told {you/SP} that {you/s/he} . . .had coronary heart disease?
Angina	MCQ160D	Ever told you had angina/angina pectoris	Has a doctor or other health professional ever told {you/SP} that {you/s/he} . . .had angina, also called angina pectoris?
Heart_Attack	MCQ160E	Ever told you had heart attack	Has a doctor or other health professional ever told {you/SP} that {you/s/he} . . .had a heart attack (also called myocardial infarction)?
Stroke	MCQ160F	Ever told you had a stroke	Has a doctor or other health professional ever told {you/SP} that {you/s/he} . . .had a stroke?
BMI	BMXBM	Body Mass Index	Body Mass Index (kg/m**2)

	I	(kg/m**2)	
Glaucoma	VIQ090	Ever told had glaucoma	{Have you/Has SP} ever been told by an eye doctor that {you have/s/he has} glaucoma (gla-co-ma), sometimes called high pressure in {your/his/her} eyes?
Smoking	SMQ040	Do you now smoke cigarettes	{Do you/Does SP} now smoke cigarettes . .
Drinking	ALQ101	Had at least 12 alcohol drinks/1 yr?	The next questions are about drinking alcoholic beverages. Included are liquor (such as whiskey or gin), beer, wine, wine coolers, and any other type of alcoholic beverage.In any one year, {have you/has SP} had at least 12 drinks of any type of alcoholic beverage? By a drink, I mean a 12 oz. beer, a 5 oz. glass of wine, or one and half ounces of liquor.
