# Supplementary material for: Absence of Association Between Glaucoma and Stroke Risk: Insights From a Cross‐Sectional Study and a Two‐Sample Mendelian Randomization Study
Source: J Ophthalmol. 2026 May 21;2026:5492641. doi: 10.1155/joph/5492641 (PMC13191766; doi:10.1155/joph/5492641)
Supplement: Supplementary file 2 — Supporting Information 2 Supporting Figure 2. Funnel plots and leave‐one‐out analysis of MR analysis. [file JOPH-2026-5492641-s006.pdf]

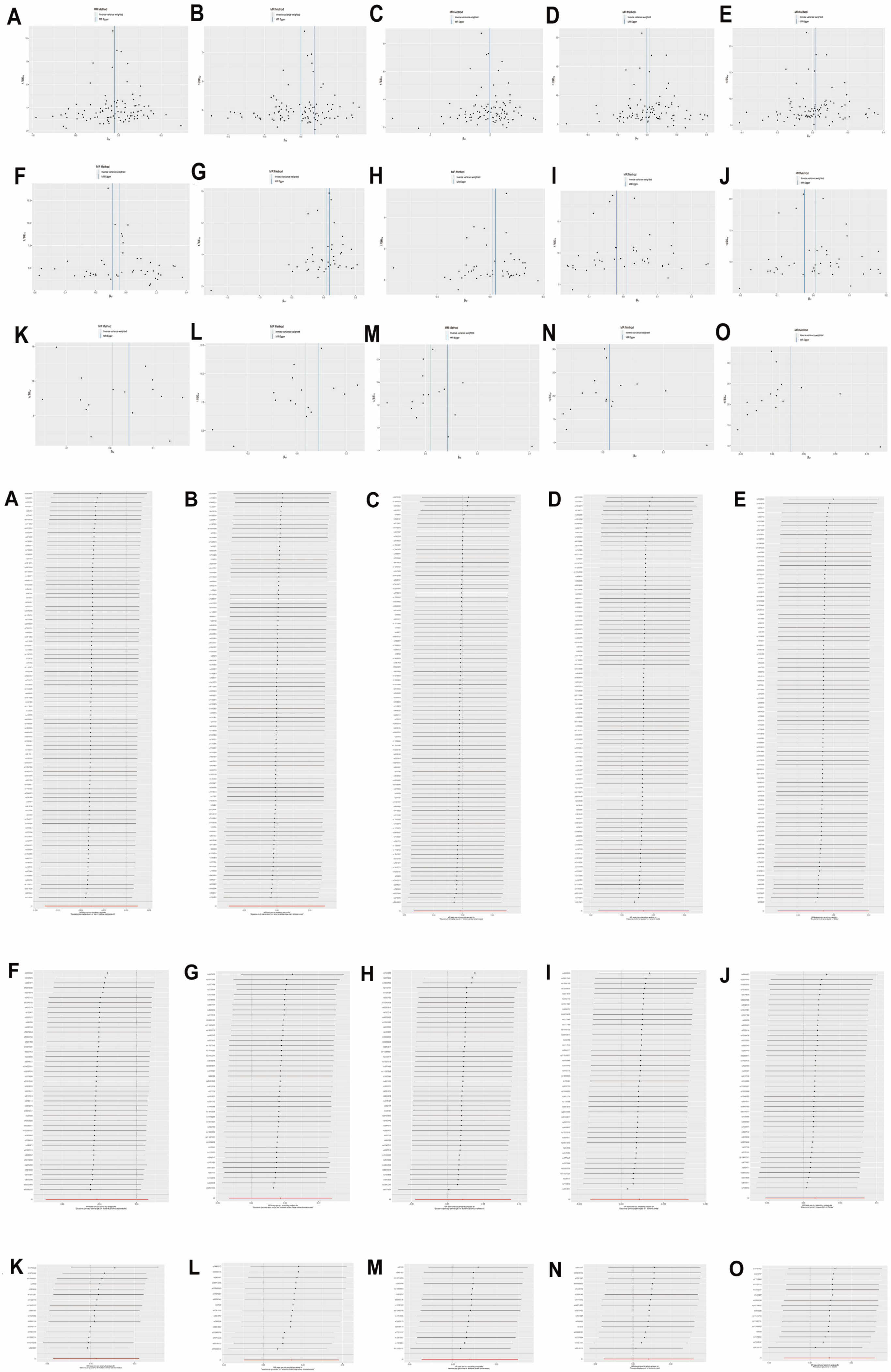
A
B
C
D
E
F
G
H
I
J
K
L
M
N
O
MR Method
Inverse variance weighted
MR Egger
